# Canada and access to medicines in developing countries: intellectual property rights first

**DOI:** 10.1186/1744-8603-9-42

**Published:** 2013-09-03

**Authors:** Joel Lexchin

**Affiliations:** 1 School of Health Policy and Management, York University, Toronto, ON, Canada; 2 Emergency Physician, University Health Network, Toronto, ON, Canada; 3 Department of Family and Community Medicine, University of Toronto, Toronto, ON, Canada

**Keywords:** Access, Anti-Counterfeiting Trade Agreement, Canada, Canada’s Access to Medicines Regime, Doha Declaration, Intellectual property rights, Least developed countries, Non-communicable diseases, TRIPS

## Abstract

Canadian reports have recommended that health as a human right must be Canada’s overarching global commitment and that the primacy of human rights should be prioritized over other elements of international law including international trade and investment law as it applies to access to pharmaceuticals. This paper uses a series of case reports to examine Canada’s commitment to this goal. Specifically it examines cases where improved access has been in conflict with increased intellectual property rights. The 6 cases are: Canada’s position when 39 pharmaceutical companies took South Africa to court in 1998 over its legislation to allow parallel importation of patented medicines and to regulate the price of medications; the stance that Canada took in the negotiations around the Doha Declaration in 2001; the passage of Canada’s Access to Medicines Regime in 2004 and subsequent attempts to amend the legislation in 2011 and 2012; Canada’s involvement in the final declaration at the United Nations High-Level meeting on non-communicable diseases in 2012; Canada’s views about the terms in the Anti-Counterfeiting Trade Agreement as expressed in 2009; and Canada’s 2013 position on the extension of the exemption for least developed countries from having to comply with the terms of the Trade Related Aspects of Intellectual Property Rights Agreement. In the first case Canada was neutral but in the remaining 5 cases Canada prioritized intellectual property rights over access. This position is consistent with how Canada has acted around domestic issues involving intellectual property rights for pharmaceutical products. Canada has supported strengthened rights despite the fact that their touted benefits have not been realized either domestically or in developing countries. As a result Canada has failed in its humanitarian duty to protect the human right to health in the form of safe and low cost medicines for the people in developing countries.

## Background

Mean per capita expenditure on pharmaceuticals in low-income countries is just over US $20 per annum, [1] and in some of these countries purchasing basic medications such as salbutamol for asthma or the antibiotic amoxicillin would push up to 86% of the population of the country below a daily income of USD $1.25 [2]. Average public sector availability of even low cost generic medicines ranges from just 29.4% to 54.4% across 36 low- and middle-income countries [3]. Under these circumstances it would seem that it’s a moral imperative for Canada to strongly support improved access to medicines in the developing world. This requirement was recognized in a report by a research consortium for the Romanow Commission, a federally appointed commission looking at the future of health care in Canada, on the subject of Canada and health care in a globalized world. In this report the researchers contended that health as a human right must be Canada’s overarching global commitment and the primacy of human rights should be prioritized over other elements of international law, including international trade and investment law as it applies to access to pharmaceuticals [4]. In his final report Romanow was quite clear that “it is time for Canada to use both its positive relationship with developing countries and its considerable expertise in health care to help improve health and health care around the world” [5].

Articles 12 and 2 of the International Covenant on Economic, Social and Cultural Rights, which Canada has both signed and ratified, commits countries to recognizing “the right of everyone to the enjoyment of the highest attainable standard of physical and mental health” (Article 12) and “to take steps…through international assistance and co-operation…with a view to achieving progressively the full realization of the rights recognized in the present Covenant” (Article 2) [6].

Finally, in the past Canada used patent legislation to protect its own population from high drug costs. A series of government reports in the 1960s found that Canadian drug prices were among the highest in the world and identified the monopoly granted by patents as being the primary reason for the prices. In response, Canada modified its patent act to allow for compulsory licensing to import generic products. The result was a significant expansion of the domestic generic industry and a substantial overall saving in the cost of prescription drugs [7]. Given its own past history Canada might have been expected to be sympathetic to the need for developing countries to provide their people with affordable medications.

This paper uses 6 case studies to explore whether this aspirational goal of helping developing countries to improve health and healthcare has actually been reflected in Canadian policy and examines the political ideology driving the decisions that Canada has made. Whether these decisions have had an effect on international organizations such as the World Trade Organization or with respect to what other countries have done is outside the scope of this paper. Specifically this article examines instances where improved access to safe and low cost drugs in developing countries has come into conflict with support for intellectual property rights (IPRs). These instances were identified by reading material on the following web sites: Canadian HIV/AIDS Legal Network <http://www.aidslaw.ca/EN/>, Health Action International <http://www.haiweb.org>, Knowledge Ecology International <keionline.org>, Oxfam Canada <http://www.oxfam.ca>, Oxfam International <http://www.oxfam.org>, Pharmaceutical Policy Research Collaboration <http://www.pharmaceuticalpolicy.ca>, Universities Allied for Essential Medicines <uaem.org>, Wemos <http://www.wemos.nl/Eng/>, World Health Organization (Intellectual Property) <http://www.who.int/topics/intellectual_property/en/> and World Trade Organization (TRIPS) <http://www.wto.org/english/tratop_e/trips_e/trips_e.htm>. The paper concludes by examining whether support for IPRs is justified given the lack of evidence of their positive benefits for developing countries.

## Discussion

### South Africa versus the drug companies

With the culmination of the Uruguay Round of trade negotiations in 1994, the World Trade Organization (WTO) came into existence on January 1, 1995 and with it the Trade Related Aspects of Intellectual Property Rights (TRIPS) Agreement, one of the 3 core agreements overseen by the organization. (The other two are the General Agreement on Trade in Services and the General Agreement on Trade and Tariffs.) All WTO member states must agree to abide by the terms of these 3 agreements. The TRIPS Agreement harmonized patent terms world-wide for a minimum of 20 years from the time that the patent application was made and mandated the granting of patents in all fields of technology including pharmaceuticals. Before TRIPS many developing countries either did not grant patents for pharmaceuticals or only granted patents on the process used to make the medication [8]. As there are usually many ways of making a medication, lower cost generic versions of medications were quickly available in countries with an active generic industry like India and Indian generics were then available for export to countries that did not grant patents on pharmaceuticals [9]. Following TRIPS, this pathway to making generics available was going to be severely curtailed by 2005, the time when developing countries had to accede to TRIPS. As a result, as the AIDS crisis exploded, by 2000 many of these countries were confronting a situation where the price for triple therapy for HIV was greater than USD $10,000 per person per year and the ability to access low cost generics was going to disappear in the near future [10].

Faced with increasing rates of HIV infection and these prices for HIV treatment, in the late 1990s the South African government passed the Medicines and Related Substances Control Amendment Act that allowed for generic substitution of off-patent medicines, transparent pricing for all medicines, and the parallel importation of patented medicines. In response, in 1998 39 multinational pharmaceutical companies, with the support of the United States (US) government and the European Commission, took the South African government to court alleging that the legislation violated both the TRIPS Agreement and the country’s constitution. Eventually, in the face of widespread public opposition the US government withdrew its support for the court case and without the US support the companies dropped their lawsuit [11]. Canada’s position was that intellectual property should be “a balance between the need to provide incentives to spur innovation and the benefits derived by society to have maximum access to new creations” [12]. Canada did not support the US but it also did not affirm the right of the South African government to take the steps that it did [4]. Although the position that Canada took during this dispute had little to do with its ultimate outcome, the position is indicative of Canada’s ambivalent stance between what should happen in the domestic and international spheres. At the same time that Canada was staying neutral in the South African case, it was also defending Canadian legislation, arguing that provisions in the TRIPS agreement “call for a liberal interpretation…so that governments would have the necessary flexibility to adjust patent rights to maintain the desired balance with other important national policies” [13]. This dichotomy between domestic and international positions is further highlighted in the next section.

### Canada and the Doha Declaration

In the preparations for the WTO Ministerial meeting in Doha in November 2001, Canada sided with Australia, Japan, Switzerland and the US in opposing a proposed resolution that would affirm that nothing in the TRIPS Agreement prevents WTO members from adopting measures to protect public health [4]. During the actual meeting Canada did reverse its position and ended up supporting the Doha Declaration that prioritized public health over trade. Some observers contend that the Canadian attitude changed in order to avoid international embarrassment. A month earlier, Canada had threatened to violate Bayer’s intellectual property rights if the company was unable to provide enough ciprofloxacin to protect the Canadian population in the event of a widespread anthrax attack and demanded a price reduction in ciprofloxacin from Bayer [14].

One of the key provisions of the Doha Declaration was that it reaffirmed the article in TRIPS stating that countries could issue compulsory licenses for medications. Additionally, the Doha Declaration made it clear that in emergencies or other circumstances of extreme urgency, countries could choose to waive the normal requirement to first attempt to negotiate a voluntary license on reasonable terms and conditions for a reasonable period of time. A compulsory license allows for the production of generic versions of a medication even while the product is still protected by patents. However, generic production had to be predominantly for domestic consumption leaving the problem of providing low cost generic medicines for countries that lacked the facilities for domestic generic manufacturing. It took another two years of difficult negotiations before the WTO members, meeting in the WTO General Council, reached an agreement, the so-called “August 30, 2003” decision, on how to resolve this issue [8]. In the run-up to this final resolution, developed countries offered two significantly different proposals. The European Union (EU) proposed a solution that would provide limited exceptions to patent rules that hindered the export of drugs under a compulsory license while the US backed a time-limited, conditional moratorium on WTO challenges to such exports. In general, developing countries saw the EU initiative as a positive step although there were certain elements that they were concerned about, whereas the reaction to the US proposal was generally negative since it didn’t offer a permanent solution to the problem. Canada backed the much more restrictive US position over the one put forward by the EU [15] and maintained its backing for months as negotiations progressed.

### Canada’s Access to Medicines Regime

Once the compromise around compulsory licensing was reached, Canada passed Bill C-9 in May 2004 thereby becoming the first country to pass legislation allowing for the production and export of generic drugs to countries that lacked their own manufacturing capacity. The chain of events that triggered this legislation included a speech in September 2003 by Stephen Lewis, then the United Nations special envoy to Africa for HIV/AIDS, and the desire of Jean Chretien, then prime minister of Canada, to leave a legacy [16]. (The legislation, now Canada’s Access to Medicines Regime (CAMR), was initially called the Jean Chretien Pledge to Africa.) However, the compromises in the legislation made the act largely unworkable. “Policy debates were dominated by two themes overall: intellectual property rights and TRIPS compliance…With the Departments of Industry Canada and International Trade as the lead institutions, the goals of protecting intellectual property and ensuring good trade relations with the United States appear to have taken priority over encouraging generic competition to achieve drug affordability” [17]. Among the flaws in the legislation were: the limited list of pharmaceutical products that were eligible for export, limitations on what countries a drug could be exported to, a short duration for a compulsory license authorizing the export of a generic, significant administrative roadblocks, a compulsory license could only be issued after advance disclosure to the patent-holder of the name of the proposed recipient country, a fixed “maximum quantity” of the product to be exported in generic form and the fact that “a generic manufacturer had to file a [separate] application for every drug, for every amount produced and for every country to which it wanted to export a drug” [18].

Built into the legislation was the requirement for a review after three years. By April 2007 when the review took place, the Conservatives had replaced the Liberals as the government of Canada. At this time, CAMR had never been employed. (Subsequently, the legislation was used to send a shipment of antiretroviral drugs to Rwanda [19]). In the initial Parliamentary debates in 2004 about CAMR there was almost no mention about promoting the right to health through access to essential medicines or the need to deal with neglected diseases, and the absence of these themes carried over to the Parliamentary review [17]. While the Liberal government was primarily focused on protecting IPRs, it was not aggressively pushing this stance in its foreign policy dealings with developing countries. This position changed under the Conservatives, as their view was that IPRs were taking on an increased importance in the knowledge economy and, according to Esmail and Kohler, under them Canada's foreign policy became much more favorable to IPRs. As one example of the more aggressive policy “the Department of Foreign Affairs and International Trade…announced in 2007 that it was assessing its interests in protecting intellectual property as it initiated trade agreements in Peru, Colombia and the Dominican Republic” [17].

As a result of this stance, the conclusion of the review was not, unexpectedly, that no amendments would be made to CAMR to improve its functionality. This conclusion was reached despite the 2006 pronouncement by Tony Clement, Conservative Minister of Health, that CAMR was a flawed piece of legislation [20]. Instead the government touted a series of measures it was taking including providing tax incentives for pharmaceutical donations to developing countries, giving $100,000 to the University of Toronto’s program to improve access to pharmaceuticals in Ghana and other West African countries and its $450 million decade long African Health Systems Initiative to support country-led efforts to strengthen health systems, improve health outcomes and make progress towards the Millennium Development Goals [21]. The benefits from these government-lead initiatives would have been a good complement to an amended CAMR, a piece of legislation that would have had a relatively quick and meaningful impact on access to low-cost essential medicines, but they should not be seen as a substitute for amending CAMR. The government concluded the report by promising to monitor the situation with respect to CAMR and make amendments if necessary.

The Conservatives had a chance to act on their promise of amending the act in 2011 when a private member’s bill to amend and simplify CAMR came up for a vote. There had not been any further use of CAMR since the shipment to Rwanda and Apotex, the one generic company that had used the legislation, had stated that it would not use it again in its current form [18]. Despite passing the House of Commons, the bill was delayed by the Conservatives in the Senate until it died with the calling of an election. Tony Clement, who had switched from Health Minister to Minister of Industry, sent a highly misleading memo to Conservative members in the Senate urging them to vote against the legislation on the grounds that it “would allow drugs that have not been certified by Health Canada to be shipped ‘to unsuspecting populations, to their detriment.’ The drugs, he wrote, could be redirected to the black market with proceeds going to non-humanitarian causes such as weapons, and the shipments could run afoul of domestic laws and traditions.” He further claimed that the legislation would lead to patent holders leaving Canada and threatening Canadian research and development (R&D) [22]. The inaccuracies in what Clement wrote are well documented in a memo put out by the Canadian HIV/AIDS Legal Network and the Grandmothers Advocacy Network [23].

There was a second opportunity to amend the legislation in the fall of 2012, but with a few exceptions even the Conservatives in the House of Commons who had supported amending the legislation in 2011 yielded to the pressure from the government and the almost unanimous vote by the Conservatives lead to the defeat of the bill. The parliamentary secretary to the Defence Minister maintained that there were better ways to help people suffering from diseases in Africa and elsewhere, and once again there were charges that Conservative Members of Parliament were spreading misinformation about the effects of the bill [24].

### United Nations high-level meeting on non-communicable diseases

Over the past decade, there has been a growing recognition of the magnitude of the morbidity and mortality associated with non-communicable diseases (NCDs) not just in developed countries but also in developing countries. Aside from some regions in Africa, the global burden of disease from NCDs is greater than it is from infectious diseases [25] and almost two-thirds of the 36.1 million deaths per year from NCDs come from the poorest countries [26]. As a consequence of numbers such as these, in September 2011 the United Nations convened a High-Level meeting to come up with a strategy for how to deal with NCDs. The main outcome of the meeting was a political declaration on their prevention and control [27]. In the lead up to the meeting Canada was instrumental in trying to weaken the final declaration by, among other things, pressing for the exclusion of a pledge to support universal health care, advocating for the removal of a section about conflict-of-interest that would have limited the involvement of food and alcohol companies in developing public health policies and in not addressing trade-related barriers to global health. Although IPRs were not directly mentioned in the statement, they are intimately linked with trade as witnessed by the provisions of the TRIPS agreement and the lack of any mention about trade-related barriers could have a negative effect on the ability of developing countries to access low-cost drugs for NCDs.

### Anti-Counterfeiting Trade Agreement

There is no doubt that counterfeit drugs are a significant problem in developing countries. The World Health Organization estimates that 10% of the drugs in these countries may be counterfeit, [28] but the question is complicated because of conflicting definitions of what counterfeit means. As Attaran and colleagues [29] point out, there are four groups of drugs that are often lumped together as “counterfeits”. True counterfeits are products that are felt to be in violation of IPRs because of alleged violations of patents, trademarks or other forms of IPR. Substandard drugs are ones that unintentionally do not meet the necessary quality standards, perhaps because of impure ingredients or manufacturing problems. Unregistered medicines are those that are present in the country without the authorization of the regulatory authority, often because of theft or illegal diversion. Finally, deliberately falsified medicines are ones created with a criminal intent to violate quality standards, e.g., by using fake ingredients. The pharmaceutical industry is primarily interested in taking action against drugs that violate their IPRs, i.e., counterfeits, but for patients the main concern is with drugs that do not meet quality and safety standards, i.e., ones that will harm their health [30].

In 2006 Japan and the US started preliminary discussions about an Anti-Counterfeiting Trade Agreement (ACTA); in 2006 and 2007 these discussions were joined by Canada, the EU and Switzerland and when formal negotiations began in 2008 Australia, Korea, Mexico, Morocco, New Zealand and Singapore were also parties. However, rather than focusing on how to stop the trade in medicines that might damage people’s health, the main focus of ACTA has been on trade in medicines that violate various forms of IPRs [31]. If enacted, the various terms in ACTA could also negatively impact on access to generic drugs in developing countries. The border measures section excludes detaining products because of patent violations but civil trademark infringements are included as a ground to detain generics passing in transit, i.e., going through a third country on the way between the exporting and importing countries [32]. This concern about generics being detained is not merely theoretical. The EU already has regulations about goods in transit that are similar to those in ACTA. “In 2008 and 2009 there were at least 19 detentions by customs authorities of medicines in transit through the EU from the source country [usually India] to destinations in Latin America and elsewhere…These detentions took place under an EU regulation…that permitted action against goods infringing intellectual property rights, including goods in transit…even though the products were not patented in India or the destination country” [30]. Patents are included in the civil enforcement section of the treaty by default. Developing countries could exclude them, but there is a well-grounded fear that these countries could be pressured into adopting the default position of including patents. Finally, “ACTA puts a broad group of third parties at risk of criminal and civil enforcement measures, including injunctions, provisional measures and claims for high damages. In the trade in generics this group of third parties can potentially include suppliers of active ingredients for medicines or NGOs procuring and distributing legitimate generics for treatment” [32].

Several of these concerns were raised in June 2009 when Foreign Affairs and International Trade Canada (DFAIT) held a roundtable with various representatives of civil society about ACTA. At the meeting, officials from Canadian Border Services Agency stated that seizures at Canadian borders of generic drugs that were in transit were unlikely to happen due to Canada’s differing legislation and practices. With regards to the issue that ACTA was only intended to deal with IPR issues and not drugs that could compromise health, DFAIT’s position was that “ACTA can make a contribution to the fight against counterfeit medicines by establishing international standards for trademark enforcement, but only as a part of Canada’s broader approach.” However, Canada is not taking any initiatives to deal with substandard or deliberately falsified medicines nor has it indicated its intent to do so in any public fashion. In addition, DFAIT was opposed to the idea of removing medicines from the scope of ACTA because its view was that ACTA is a non-sectoral agreement, i.e., an agreement about counterfeiting in general, and removing pharmaceuticals would result in lower sectoral enforcement standards [33].

Although the European Parliament overwhelmingly rejected ACTA, [34] Canada has signed the agreement although it is yet to be ratified [35].

### Negotiations for TRIPS extension for least developed countries

Least developed countries (LDCs), as defined by the United Nations, are those with a gross domestic product per capita of less than USD $905, human resource weakness and economic vulnerability. Currently 49 countries have this designation [36]. When the TRIPS agreement came into force on January 1, 1995 LDCs were granted a 10-year exemption from complying with its provisions and this exemption was subsequently extended to July 1, 2013. Separately, these countries were given an extension until January 1, 2016 before they need to provide for or enforce either patents or data protection (data protection prevents generic companies from using the safety and efficacy data produced by brand-name companies for a specific period of time) for pharmaceutical products [37]. As the July 2013 deadline neared, the LDCs started lobbying for an indefinite extension that would apply until a country no longer fell into the LDC category [38]. This position echoed the recommendation from the Global Commission on HIV and the Law that “WTO Members must indefinitely extend the exemption for LDCs from the application of TRIPS provisions in the case of pharmaceutical products” [39].

The US and the EU backed by a group of developed countries including Canada, Australia, Japan, New Zealand and Switzerland were opposed to an indefinite extension and instead argued for a time limited extension of between 5 and 10 years. In addition, these countries were opposed to the elimination of the “no rollback” condition that was included in the 2005 extension [40]. This clause prevented LDCs from repealing or revising any TRIPS related IPR provisions that they had already implemented, thereby preventing LDCs from being able to experiment with IPRs that were appropriate for their level of development [37,41]. The final compromise was for an 8-year extension. Although the no rollback clause was not eliminated, LDCs will be allowed to use the “flexibilities” in TRIPS to introduce measures such as compulsory licensing, but only if they “express their determination to preserve and continue their progress towards implementing the TRIPS agreement” [42]. The EU is already interpreting this phrase in a much more restrictive manner than are the LDCs [43]. There is no similar requirement in the special exemption for pharmaceutical products and therefore, if that special exemption is not renewed, then come January 1, 2016 LDCs may have much more difficulty in being able to work around the IPR requirements in TRIPS. Canada’s position in this recent set of negotiations sets the stage for its likely stance when the question of whether to extend the 2016 exemption, and what conditions will apply to an extension, becomes an issue.

## Summary

It would be a mistake to conclude that Canada has not done anything to help improve access to medications in developing countries. Between 2001 and 2010 Canada contributed over USD $874,000,000 to the Global Fund to Fight AIDS, Tuberculosis and Malaria ranking it 7^th^ among individual country donors [44]. However, whenever there has been a conflict between access and supporting IPRs, the Canadian government regardless of its political leanings, has either been neutral as in the South African court case or consistently backed strong IPRs. The “goals of protecting intellectual property and ensuring good trade relations with the United States” have taken precedence over the need for safe, low cost medications in poor countries [17]. The supremacy of IPRs over access shows the triumph of neoliberalism, in the form of individual property rights, over collective security through access to medications.

The Canadian prioritization of IPRs internationally is a reflection of what has been happening domestically in Canada for over 25 years. The increased emphasis on IPRs by Canadian governments is matched by promises from the pharmaceutical industry about increased R&D investment contingent on stronger IPRs [45,46]. This process started with the weakening of compulsory licensing for the domestic production of generic drugs in 1987 [45] and progressed through the abolition of compulsory licensing in 1993, [47] the imposition of the Notice of Compliance regulations, also in 1993, that can delay the appearance of generic drugs for up to 24 months [48] and the 2006 extension of the data protection period to between 8 and 8.5 years [49]. The rationale behind all of these decisions has been to encourage pharmaceutical investment in R&D in Canada. However, between 2010 and 2013 seven major multinational pharmaceutical companies have closed research facilities in Canada with a loss of over 1000 jobs [50]. The disinvestment emphasizes the fact that the strength of IPRs is only one of many factors that influence decisions when it comes to direct foreign investment by pharmaceutical companies [51]. According to a study looking at the prospects for national drug insurance in Canada, the Canadian government’s stance on IPRs has lead to the country having the 3^rd^ or 4^th^ most expensive brand name products among 8 major developed countries [52]. Finally, spending on R&D by the brand-name companies is 6.7% of sales putting Canada in virtually the same position it was in in 1988 [53].

Internationally, the expectation by the Canadian government that the poorest countries in the world should adopt IPRs that are equivalent to those in wealthy developed countries ignores both historical and current economic realities. Many major western countries including Canada, Denmark, Sweden and Switzerland did not adopt full IPRs for pharmaceuticals until the 1970s and 1980s when their gross domestic product per capita was in the range of USD $16,000 to $36,000 [54]. Expecting the same from countries with a GDP of less USD $1000 is folly. In the words of the Commission on Intellectual Property Rights “It is our contention that intellectual property systems may, if we are not careful, introduce distortions that are detrimental to the interests of developing countries. Very ‘high’ standards of protection may be in the public interest in developed countries with highly sophisticated scientific and technological infrastructures…but this does not mean the same standards are appropriate in all developing countries…so far as possible developing countries should not be deprived of the flexibility to design their IP systems that developed countries enjoyed in earlier stages of their own development, and higher IP standards should not be pressed on them without a serious and objective assessment of their development impact” [55].

Despite repeated claims that strong IPRs will benefit the LDCs the opposite is true. Between 1975 and 2004 only 21 out of 1556 marketed new chemical entities were indicated for the neglected diseases that occur largely or exclusively in the LDCs [56]. In the first half of the 2000s, 5 out of 12 of the top multinational companies were not conducting any research on neglected diseases and these companies were unwilling to enter this area regardless of any incentives offered to them [57]. Speaking to a reporter from the Financial Times, Daniel Vasella, then the CEO of Novartis, said “We have no model which would (meet) the need for new drugs in a sustainable way … You can't expect for-profit organization[s] to do this on a large scale. If you want to establish a system where companies systematically invest in this kind of area, you need a different system” [58]. While patent protection in developed countries leads to more R&D in some countries, although not all as the Canadian data shows, for developing countries there is no relationship between patent protection and investment in R&D [59]. In addition, “the introduction of patents in developing countries has not been followed by greater R&D investment in the diseases that are most prevalent there” [60]. Similarly, there is no relationship between whether a country adopts data exclusivity and the amount of investment by the pharmaceutical industry in the country [61].

The decisions about expanding access to essential medicines by Canadian governments over the past decade and a half have been motivated by a free market ideology that prioritizes private property in the form of IPRs. As a result, Canada has failed in its humanitarian duty to protect the human right to health in the form of safe and low cost medicines for the people in developing countries.

## Competing interests

In 2007 Joel Lexchin was a consultant to a law firm acting for Apotex Inc. In 2008 he was an expert witness for the Canadian federal government in its defence against a lawsuit challenging the ban on direct-to-consumer advertising. In 2010 he was an expert witness for a law firm representing the family of a plaintiff who allegedly died from an adverse reaction from a product made by Allergan. He is currently on the Management Board of Healthy Skepticism Inc. and is the Chair of the Health Action International – Europe Association Board.
